# Detection and genetic characterization of *Echinococcus granulosus* mitochondrial DNA in serum and formalin-fixed paraffin embedded cyst tissue samples of cystic echinococcosis patients

**DOI:** 10.1371/journal.pone.0224501

**Published:** 2019-10-29

**Authors:** Maryam Moradi, Ahmad Reza Meamar, Lame Akhlaghi, Mona Roozbehani, Elham Razmjou

**Affiliations:** 1 Department of Parasitology and Mycology, School of Medicine, International Campus, Iran University of Medical Sciences, Tehran, Iran; 2 Department of Parasitology and Mycology, School of Medicine, Iran University of Medical Sciences, Tehran, Iran; Dokkyo Medical University, JAPAN

## Abstract

Cystic echinococcosis (CE) is a worldwide zoonotic disease caused by the larval stage of *Echinococcus granulosus*. We investigated the presence of *E*. *granulosus-*specific DNA in the serum of CE patients by detecting the cytochrome c oxidase I (*cox1*) and NADH dehydrogenase subunit I (*nad1*) mitochondrial genes. Serum and formalin-fixed paraffin embedded (FFPE) cyst tissue samples of 80 CE patients were analyzed. The extracted DNA of samples was submitted to PCR amplification of *cox1* and *nad1* genes, and products were sequenced and genotyped. Nineteen (23.8%; 95% CI 15.8–34.1) serum and 78 (97.5%; 95% CI 91.3–99.3) FFPE cyst tissue samples were successfully amplified with at least one gene. *Echinococcus* DNA was detected in the sera of 15.0% (95% CI: 8.8–24.4) and 10.0% (95% CI: 5.2–18.5) and in cyst tissue of 91.3% (95% CI: 83.0–95.7) and 83.8% (95% CI: 74.2–90.3) of 80 patients by *cox1* and *nad1* gene, respectively. Four genotypes of *E*. *granulosus* were distinguished in the CE patients, with predominance of genotype G1, followed by G3, G2, and G6. The finding of *E*. *granulosus* DNA in 23.8% of serum samples from CE patients confirmed that *E*. *granulosus* releases cell-free DNA into the circulatory system, but quantities may be inadequate for the diagnosis of CE. Genotype G1 predominance suggests the sheep-dog cycle as the primary route of human infection.

## Introduction

Cystic echinococcosis (CE), or hydatid cyst disease, is a tissue infection resulting from the development of a larval metacestode stage after ingestion of eggs of *Echinococcus granulosus* sensu lato, a complex of four species and ten genotypes classified according to the host range and genetic diversity: *E*. *granulosus* sensu stricto (G1 to G3), *Echinococcus equinus* (G4), *Echinococcus ortleppi* (G5), and *Echinococcus canadensis* (G6 to G10) [1–3]. Human infection usually occurs following ingestion of eggs in water or food contaminated with canid feces [4]. This zoonotic disease has worldwide distribution and is endemic in many countries, including Iran [5]. Human CE is reported in all parts of Iran and is the basis for nearly 1% of all surgical procedures [6] and 25% of liver and lung surgeries [7]. The condition becomes symptomatic as the cyst grows, with highly variable clinical manifestations depending on location and size [8]. Diagnosis of CE based on clinical findings is unreliable, and is usually confirmed through imaging and antibody detection [9]. Variations in antibody titer during cyst growth, as well as cross-reactions, means that hydatid antibody assessment alone may not confirm clinical diagnosis [10]. Tissue samples are a valuable source for precise molecular identification and *Echinococcus* genotyping, but this is invasive so is usually performed after cystectomy to confirm the cyst type and for confirming diagnosis by direct parasite identification from histology.

Diagnosis of early-stage CE is critical to effective drug treatment, but CE is usually only detected at the end stage, when the cyst is large and complex, and surgery is the only therapeutic option [11, 12]. Identification of *Echinococcus* DNA in patient serum may be a feasible non-invasive method of diagnosis of CE. The goal of this study was to assess detection of *E*. *granulosus*-specific DNA in CE patient serum by tracing cytochrome c oxidase I (*cox1*) and NADH dehydrogenase subunit I (*nad1*) mitochondrial genes. The serum DNA findings were compared with those of excised cysts for confirmation. The genotype and genetic diversity of positive samples were determined by sequencing of *cox1* and *nad1* genes to specify the source of DNA in the serum of CE patients.

## Material and methods

### Ethics statement

The ethics committee of Iran University of Medical Sciences approved the study protocol and informed consent arrangements [IR.IUMS.REC 1395.9223651201]. Patients were informed of the study objectives and gave written informed consent for their blood and tissue samples to be used for research.

### Sample collection and histology

Serum and cyst tissue samples of 80 patients who had undergone echinococcosis cyst removal surgery in Milad Hospital, Tehran, from April 2015 to December 2017, were included in the study. After radical surgery, cyst tissue samples were fixed in 10% formalin. Macroscopic observations were recorded, and samples were embedded in paraffin according to routine histological procedures. Five μm sections were stained with hematoxylin and eosin and examined by light microscopy.

### DNA extraction and polymerase chain reaction

The DNA was extracted from 200 μl of serum by QIAamp DNA Blood Minikit (Qiagen, Germany) according to the manufacturer’s instructions. Five 10 μm sections were cut from each embedded cyst tissue sample, and excess paraffin was trimmed. The prepared sections were submitted to the DNA extraction procedure of GeneRead DNA FFPE Tissue Kit (Qiagen, Germany) according to manufacturer’s instructions. The obtained genomic DNA of cyst and serum samples was stored at -20°C until analysis.

The DNA of *E*. *granulosus* was detected by PCR amplification of two mitochondrial genes, *cox1* and *nad1*, in patient sera and cysts. The 400 bp fragments of *cox1* and 450 bp of *nad1* genes were amplified by primers as described by Bowles [13] and Sharbatkhori [14], respectively. The final mixture of the PCR reaction contained 25 μl of Taq DNA Polymerase Master Mix (2X) (Amplicon III, Denmark, Cat no. 180301), 0.5 μM of each primer, 3–5 μl DNA. PCR was conducted under the conditions: 94°C for 5 min initial denaturation; 35 cycles of 94°C for 45 s, 55°C for 30 s, 72°C for 35 s; and a final extension at 72°C for 5 min. PCR products were visualized on 1.5% agarose gel. To validate accuracy of PCR results, DNA extracted from the laminated layer of a hydatid cyst and distilled water were used as positive and negative controls, respectively, and processed with the samples in each PCR set.

### Sequencing and phylogenetic analyses

PCR products were purified from agarose gel using the MinElute gel extraction kit (QIAGEN Ltd., Hilden, Germany), sequenced in both directions using forward and reverse primers (Macrogen Inc., Seoul, South Korea), and read by Chromas software (Technelysium Pty Ltd., Queensland, Australia). The forward and reverse sequences of each sample were aligned and assembled using DNASIS MAX (version 3.0; Hitachi, Yokohama, Japan) and BLAST searched (http://blast.ncbi.nlm.nih.gov) to compare similarity with sequences in GenBank database. Sequences were deposited in GenBank under accession numbers LC476594–LC476659 for *cox1* and LC476660–LC476714 for *nad1*. The final sequences of each samples were aligned with reference sequences for each genotype to determine *E*. *granulosus* genotype in MEGA 7 (www.megasoftware.net). A concatenated sequence of each sample was obtained by combining the *cox1* and *nad1* sequences. The phylogenetic tree was created with MEGA 7 software using neighbor-joining algorithms with evolutionary distances calculated by the Kimura-2 parameter method and a bootstrap value of 1000.

## Results

### Demographic characteristics of patients

The 80 participants comprised 28 (35%) males and 52 (65%) females, aged 7 to 76 years with a mean of 39 years. The largest number of subjects fell into the 31–45 year age range with the fewest in the ≤15 years category (Table 1). Cyst location was primarily liver (70%), followed by lung (22.5%), with rare cases in kidney, brain, common bile duct, and omentum (1.3%) (Table 1). One patient had cysts in both liver and lung, and another in liver and spleen.

**Table 1:** Demographic data of patients undergoing cystic echinococcosis surgery, histological features of the removed cyst, and molecular identification of *Echinococcus granulosus* in formalin-fixed paraffin embedded cyst tissue and serum by the cytochrome c oxidase I (*cox1*) and NADH dehydrogenase subunit I (*nad1*) mitochondrial genes.

Variable	Organ No. (%)						Total No. (%)
	Liver	Lung	Kidney	Brain	Bile duct	Omentum	
Sex							
Male	22 (75.0)	5 (17.9)	0	0	0	1 (3.6)	28 (35.0)
Female	36 (67.3)	13 (25.0)	1 (1.9)	1 (1.9)	1 (1.9)	0	52 (65.0)
Total	58 (72.5)	18 (22.5)	1 (1.3)	1 (1.3)	1 (1.3)	1 (1.3)	80 (100.0)
Age years							
0–15	1	1	0	1	0	0	3 (3.8)
16–30	12	7	0	0	1	0	20 (25.0)
31–45	28	7	0	0	0	0	35 (43.8)
46–60	14	2	0	0	0	0	16 (20.0)
>60	3	1	1	0	0	1	6 (7.5)
Total cases	58	18	1	1	1	1	80 (100)
Cyst dimensions (cm)	7.8×6	7.5×5.6	2×1	9×7	3×3	6×6	
Wall thickness (cm)	1.4	2.1	1.0	0.2	1.0	1.5	
*Cox1*							
Tissue	51	18	1	1	1	1	73 (91.3)
Serum	10	2	Na	na	na	na	12
*Nad1*							
Tissue	46	17	1	1	1	1	67 (83.8)
Serum	7	1	Na	na	na	na	8

na: not amplified.

### Histology

**Fig 1:**
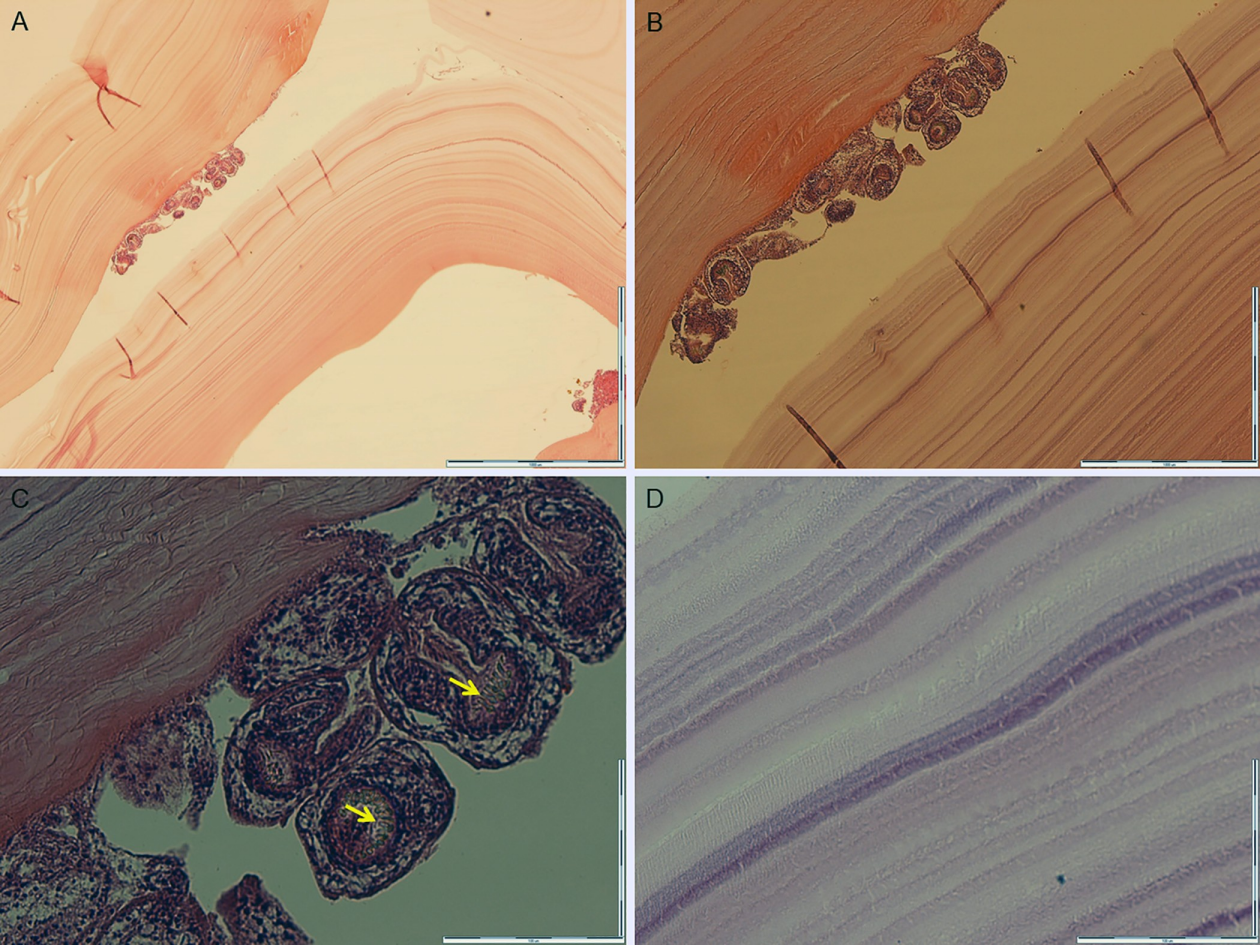
Formalin-fixed paraffin embedded *Echinococcus granulosus* cyst stained with hematoxylin and eosin. Cross-section of an *Echinococcus granulosus* cyst, the cyst wall and protoscoleces (A) 40×, (B) 100× and (C) 400× magnification. Arrows indicate the hooklets inside the protoscoleces D) Acellular laminated layers of the cyst wall 1000× magnification.

The cyst dimensions and wall thickness were recorded (Table 1). Length of liver and lung cysts ranged from 1 to 25 cm and 3 to 18 cm, respectively. The existence of laminated layers, protoscoleces, or hooklets of *E*. *granulosus* in cysts confirmed CE (Fig 1).

### Molecular analysis

The *cox1* and *nad1* genes were amplified in 73 and 67 of FFPE cyst tissue samples and in 12 and 8 serum samples, respectively (n = 80) (Table 1). Nineteen (23.8%; 95% CI 15.8–34.1) of serum and 78 (97.5%; 95% CI 91.3–99.3) of cyst tissue samples were successfully amplified with at least one gene.

The *cox1* fragments were successfully sequenced in 59 FFPE cyst and 11 serum samples, and *nad1* in 51 FFPE cyst and eight serum samples (Table 2). The genotype determined in serum samples was identical with that identified in the corresponding cyst tissues. The G1 genotype was identified in 50 of 59 *cox1* and 36 of 51 *nad1* fragments. BLAST search identified two samples as G6 genotype with both *cox1* and *nad1* (Table 2). Three samples were identified as G2 genotype with *cox1*, with *nad1* two of these samples showed 100% identity to G2 (AJ237633) or G3 (AJ237634 and FJ796214) genotype sequences, so they were designated as G2/G3. One sample was not successfully sequenced. Of thirteen samples identified as G2/G3 genotypes with *nad1*, *cox1* determined ten samples as G1, two as G2, and one as G3.

**Table 2:** Genotypes of *Echinococcus granulosus*, identified in formalin-fixed paraffin embedded cyst tissue and serum by the cytochrome c oxidase I (*cox1*) and NADH dehydrogenase subunit I (*nad1*) mitochondrial genes.

Organ	Genotype						
	*cox1*				*nad1*			
	G1	G2	G3	G6	G1	G2/3	G6
Liver							
Tissue	35	3	4	1	24	10	1
Serum	9	0	0	0	6	1	0
Lung							
Tissue	11	0	0	1	10	2	1
Serum	2	0	0	0	1	0	0
Kidney							
Tissue	1	0	0	0	1	0	0
Serum	0	0	0	0	0	0	0
Brain							
Tissue	1	0	0	0	1	0	0
Serum	0	0	0	0	0	0	0
Bile Duct							
Tissue	1	0	0	0	0	1	0
Serum	0	0	0	0	0	0	0
Omentum							
Tissue	1	0	0	0	0	0	0
Serum	0	0	0	0	0	0	0
Total							
Tissue	50	3	4	2	36	13	2
Serum	11	0	0	0	7	1	0

Tehran Province contributed the highest number of participants to this study. The genotype distribution according to the patient province of residency is shown in Table 3 and S1 Table.

**Table 3:** Genotype distribution of *Echinococcus granulosus* identified by the cytochrome c oxidase I (*cox1*) and NADH dehydrogenase subunit I (*nad1*) mitochondrial genes according to the patient province of residency.

Province	No. (%)	Genotype No. (%)						
		*cox1*				*nad1*		
		G1	G2	G3	G6	G1	G2/3	G6
Tehran	48 (73.8)	37 (74.0)	2 (66.7)	2 (50.0)	1 (50.0)	29 (80.6)	8 (61.5)	1 (50.0)
Alborz	5 (7.7)	4 (8.0)	1 (33.3)	0 (0.0)	0 (0.0)	2 (5.6)	2 (15.4)	0 (0.0)
Kurdistan	1 (1.5)	1 (2.0)	0 (0.0)	0 (0.0)	0 (0.0)	0 (0.0)	1(7.7)	0 (0.0)
Markazi	3 (4.6)	3 (6.0)	0 (0.0)	1 (25.0)	0 (0.0)	2 (5.6)	0 (0.0)	0 (0.0)
East Azerbaijan	2 (3.1)	1 (2.0)	0 (0.0)	0 (0.0)	0 (0.0)	2 (5.6)	0 (0.0)	0 (0.0)
West Azerbaijan	1 (1.5)	1 (2.0)	0 (0.0)	0 (0.0)	0 (0.0)	1 (2.8)	0 (0.0)	0 (0.0)
Ilam	1 (1.5)	1 (2.0)	0 (0.0)	0 (0.0)	0 (0.0)	0 (0.0)	1 (7.7)	0 (0.0)
Zanjan	1 (1.5)	1 (2.0)	0 (0.0)	0 (0.0)	0 (0.0)	0 (0.0)	0 (0.0)	0 (0.0)
Qazvin	2 (3.1)	1 (2.0)	0 (0.0)	1 (25.0)	0 (0.0)	0 (0.0)	1 (7.7)	0 (0.0)
Mazandaran	1 (1.5)	0 (0.0)	0 (0.0)	0 (0.0)	1 (50.0)	0 (0.0)	0 (0.0)	1 (50.0)
Total	65 (100.0)	50 (100.0)	3 (100.0)	4 (100.0)	2 (100.0)	36 (100.0)	13 (100.0)	2 (100.0)

**Fig 2:**
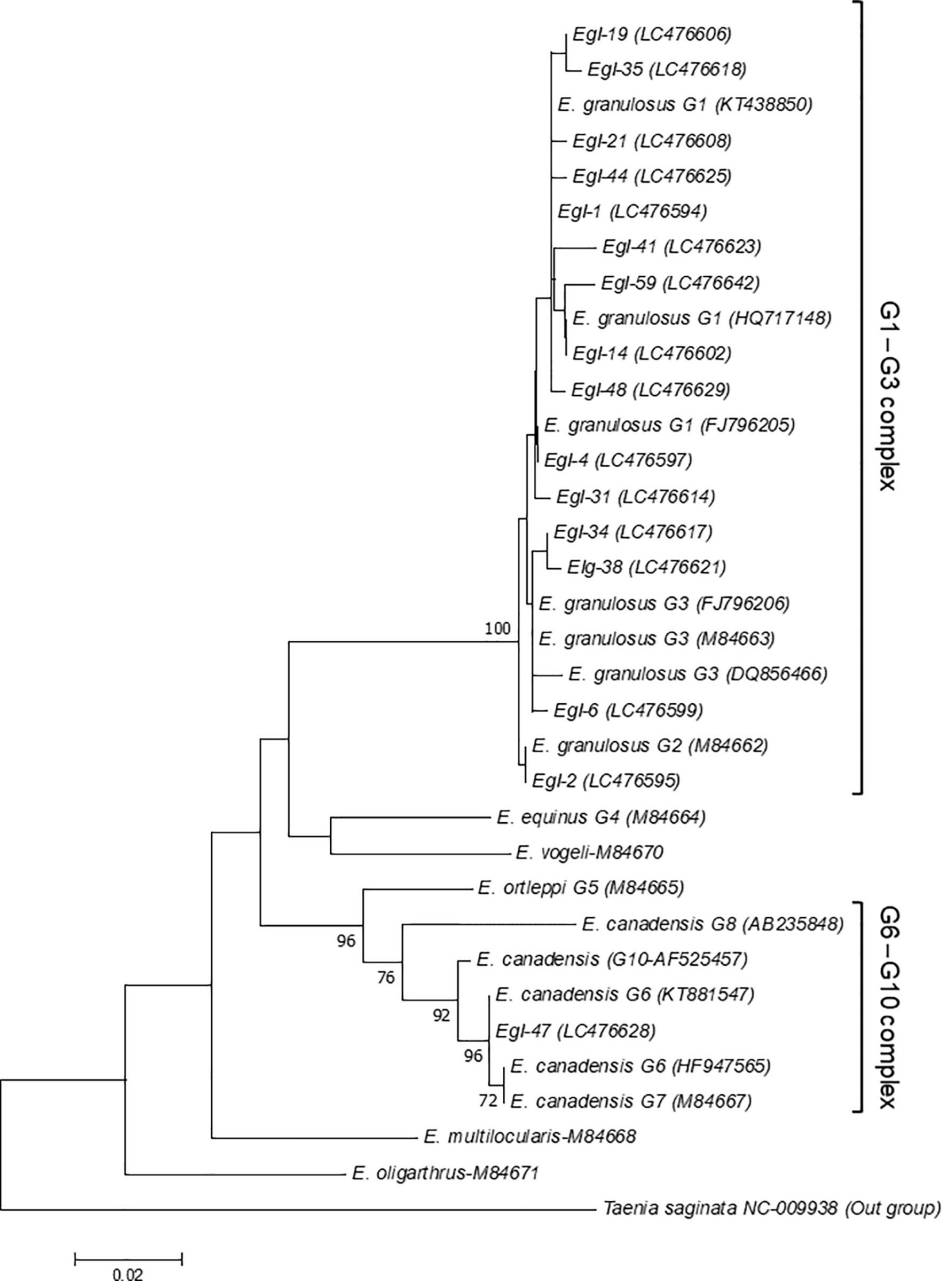
Phylogram of *Echinococcus granulosus* sensu lato was inferred based on the nucleotide sequences of the cytochrome c oxidase I gene (*cox1*). The evolutionary relationship of *Echinococcus granulosus* sensu lato was constructed by the neighbor-joining method, based on the nucleotide sequences of *cox1* retrieved from this study (S1 Table) compared with reference sequences of *E*. *granulosus* sensu lato and other species of *Echinococcus* from GenBank, with *Taenia saginata* as outgroup. Bootstrap values obtained from 1000 replicates are indicated on branches in percentage, and only bootstraps values >70% are displayed. Evolutionary analyses were conducted in MEGA7.

The *cox1* sequencing multiple alignments of 50 G1-genotype isolates were grouped into 11 patterns according to the single nucleotide polymorphisms of isolates compared with published sequences for the G1 genotype (Table 4). Twenty-nine isolates showed 100% homology with published G1 sequence KT438850, three with HQ717148, and two with FJ796205. The remaining eight sequence patterns showed one to three nucleotide substitutions with the G1 genotype GenBank sequences KT438850, HQ717148, FJ796205, and DQ856467 (Table 4). Three samples of the G2 genotype showed complete identity with *cox1* reported sequence M84662. Four isolates grouped in three patterns showing one or two substitutions relative to the G3 genotype GenBank sequence M84663. Two samples showed 99% identity to the G6 genotype sequence HF947565, with a single nucleotide substitution of C for T at position 40. Phylogenetic analysis of *cox1* supported the alignment of 15 patterns classified as G1-G3 complex with 100% bootstrap value; one pattern grouped in G6-G10 complex with high bootstrap value (Fig 2).

**Table 4:** Multiple alignment sequences of cytochrome c oxidase I (*cox1*) gene obtained from formalin-fixed paraffin embedded hydatid cyst tissue and patient serum with reference sequences retrieved from GenBank.

Isolate/GenBank no.	Nucleotide position																			
**Genotype 1**	3	6	7	10	29	30	37	40	56	66	122	157	173	181	203	204	228	231	246	257
KT438850	T	A	T	G	G	T	A	A	C	C	G	A	G	A	C	T	A	T	G	T
HM636641	.	.	.	.	.	.	.	.	.	.	.	.	.	.	.	.	.	.	.	.
FJ796205	.	.	.	.	.	.	.	.	.	T	.	.	.	.	.	.	.	.	.	
HQ717148	.	.	.	.	.	.	.	.	T	.	.	.	.	.	.	.	.	.	.	.
DQ856467	.	.	.	.	.	.	.	.	.	T	.	.	.	.	.	.	.	.	.	C
EgI-1, 3, 5, 8, 15, 18, 20, 22–25, 32, 33, 36, 37, 45, 46, 51,54–57, 61, 62, 66, 69, 70, 73, 75, 51s, 54s, 56s, 57s, 70s	.	.	.	.	.	.	.	.	.	.	.	.	.	.	.	.	.	.	.	.
EgI-4, 39	.	.	.	.	.	.	.	.	.	T	.	.	.	.	.	.	.	.	.	.
EgI-14, 60, 67	.	.	.	.	.	.	.	.	T	.	.	.	.	.	.	.	.	.	.	.
EgI-19, 29, 42	.	.	.	.	.	.	.	.	.	.	.	.	.	.	.	.	.	C	.	.
EgI-21	.	.	.	.	.	.	.	.	.	.	.	.	.	.	.	.	.	.	.	C
EgI-31	.	.	.	.	.	.	.	G	.	.	.	.	.	.	.	.	.	.	.	.
EgI-35	.	.	.	.	.	.	.	.	.	.	T	.	.	.	.	.	.	C	.	.
EgI-41	.	.	C	.	A	.	G	.	.	.	.	.	.	.	.	.	.	.	.	.
EgI-44	.	.	.	.	.	.	.	.	.	.	.	G	.	.	.	.	.	.	.	.
EgI-48, 49, 50, 68, 72, 74, 78, 68s, 74s	.	.	.	.	.	.	.	.	.	.	.	.	.	.	.	.	G	.	.	.
EgI-59	.	C	A	.	.	.	.	.		.	.	.	.	.	.	.	.	.	.	.
**Genotype 2**	56	162	204																	
M84662	T	T	T																	
EgI-2, 17, 65	.	.	.																	
**Genotype 3**	15	30	53																	
M84663	T	T	A																	
EgI-6, 7	C	C.	.																	
EgI-34	C	.																		
EgI-38	C	.	G																	
**Genotype 6**	40																			
HF947565	C																			
EgI-47, 53	T																			

Egl-n: *Echinococcus granulosus* isolate.

**Fig 3:**
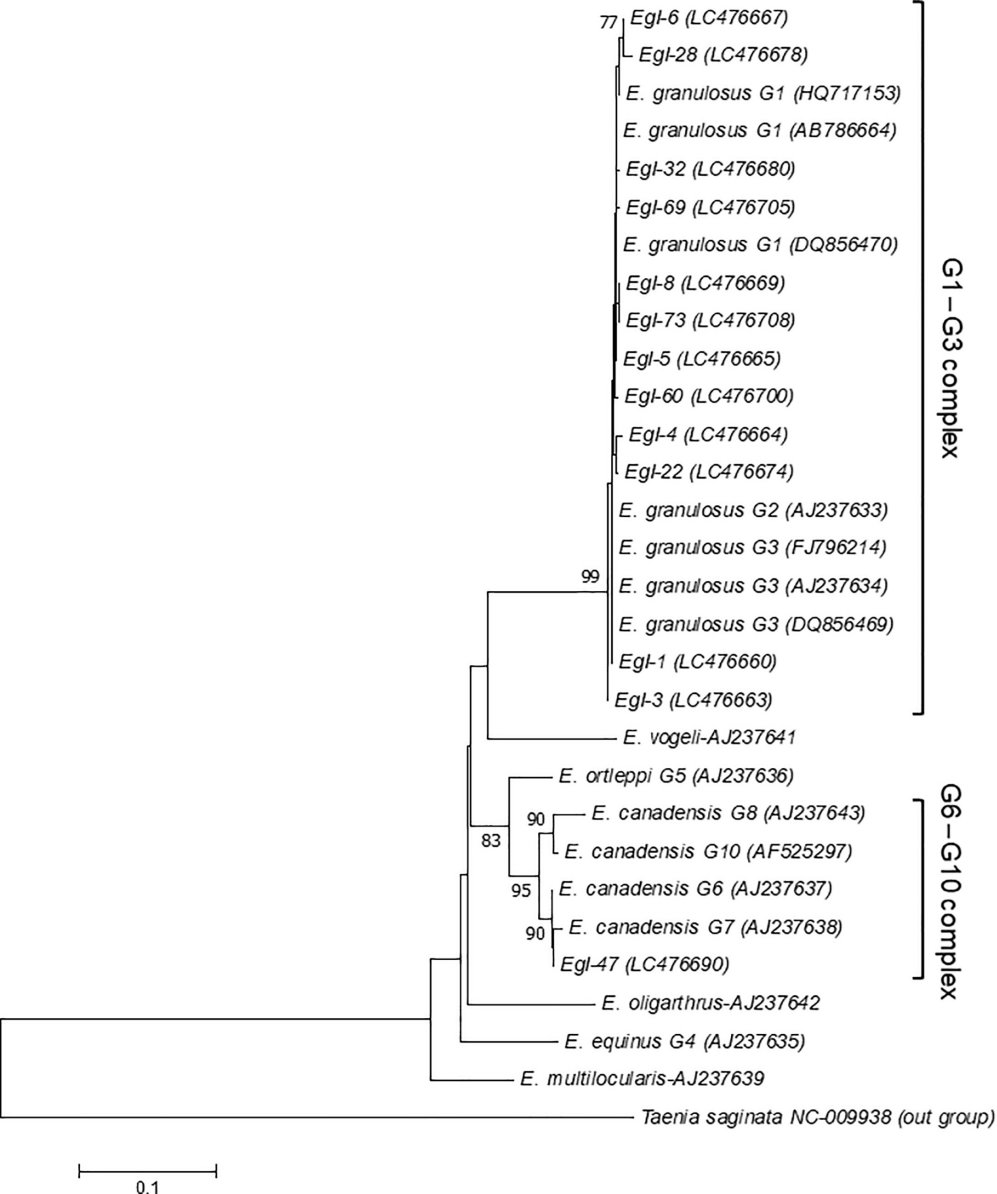
Phylogram of *Echinococcus granulosus* sensu lato was inferred based on the nucleotide sequences of the NADH dehydrogenase subunit I gene (*nad1*). The evolutionary relationship of *Echinococcus granulosus* sensu lato was constructed by the neighbor-joining method, based on the nucleotide sequences of *nad1* retrieved from this study (S1 Table) compared with reference sequences of *E*. *granulosus* sensu lato and other species of *Echinococcus* from GenBank, with *Taenia saginata* as outgroup. Bootstrap values obtained from 1000 replicates are indicated on branches in percentage, and only bootstraps values >70% are displayed. Evolutionary analyses were conducted in MEGA7.

The alignment of *nad1* sequences of 36 G1 genotype isolates showed nine patterns, of which eight showed one to three nucleotide substitutions compared to published G1 genotype sequences (Table 5). Twenty-three isolates had 100% identity with published G1 sequence DQ856470. Six samples showed 100% identity to G2/G3 genotype sequences AJ237633/ AJ237634, and FJ796214; and seven samples showed one or two nucleotide substitutions (Table 5). Sequencing of two samples revealed 100% identity with the G6 HM636642 reference. The sequencing pattern distribution in *nad1* alignment was depicted in the phylogenetic tree. Twelve patterns clustered with the G1-G3 complex and one pattern with the G6-G10 complex with high bootstrap value (Fig 3).

**Table 5:** Multiple alignment sequences of NADH dehydrogenase subunit I (*nad1*) in formalin-fixed paraffin embedded hydatid cyst tissue and patient serum with reference sequences retrieved from GenBank.

Isolate/GenBank no.	Nucleotide position																						
**Genotype 1**	18	24	25	37	45	54	55	61	62	78	80	106	150	187	268	280	284	286	317	333	334	336	338
HQ717153	T	G	A	T	T	T	T	G	C	C	C	A	C	C	T	T	T	T	T	T	A	G	A
DQ856470	.	.	.	.	.	.	.	.	.	.	.	.	.	T	.	.	.	.	.	.	.	.	.
AB786664	.	.	.	.	.	.	.	.	.	.	.	.	.	T	.		.	.	.	.	.	.	.
EgI-5, 14, 26, 29, 33, 35,37, 38, 42, 45, 46, 49, 50, 52, 55, 59, 61, 72, 75, 76, 77, 78, 79, 5s, 37s, 59s	.	.	.	.	.	.	.	.	.	.	.	.	.	T	.	.	.	.	.	.	.	.	.
EgI-6, 36	.	.	.	.	.	.	C	.	.	.	.	.	.	.	.	.	.	.	.	.	.	.	.
EgI-8, 21, 23	.	.	.	.	.	.	.	.	.	.	.	.	.	T	.		.	C	.	.	.	.	.
EgI-22, 74	-	-	-	-		.	.	A	.	.	.	.	.	T	.	.	.	.	.	.	.	.	.
EgI-28	.	.	.	.	.	.	C	.	.	.	.	.	.	.	.	.	C	C	-	-	-	-	-
EgI-32, 54	.	.	.	.	.	C	.	.	.	.	.	.	.	T	.	.	.	.	.	-	-	-	-
EgI-60	.	.	.	.	.	.	.	.	T	.	.	.	.	T	.	.	.	.	.	.	.	.	
EgI-69	C	.	.	.	.	.	.	.	.	.	.	.	.	T	.	.	-	-	-	-	-	-	-
EgI-73	.	.	.	.	.	.	.	.	.	.	.	.	.	T	.	C	.	.	.	.	.	.	.
**Genotype 2/3**	54	61	149	187																			
FJ796214	T	G	C	T																			
EgI-1, 2, 7, 24, 57, 70,1s	.	.	.	.																			
EgI-3,15,19, 65, 66, 68	.	.	T	.																			
EgI-4	.	A	.	.																			
**Genotype 6**	68	77	109																				
HM636642	T	T	A																				
EgI-47, 53	.	.	.																				

Egl-n: *Echinococcus granulosus* isolate.

**Fig 4:**
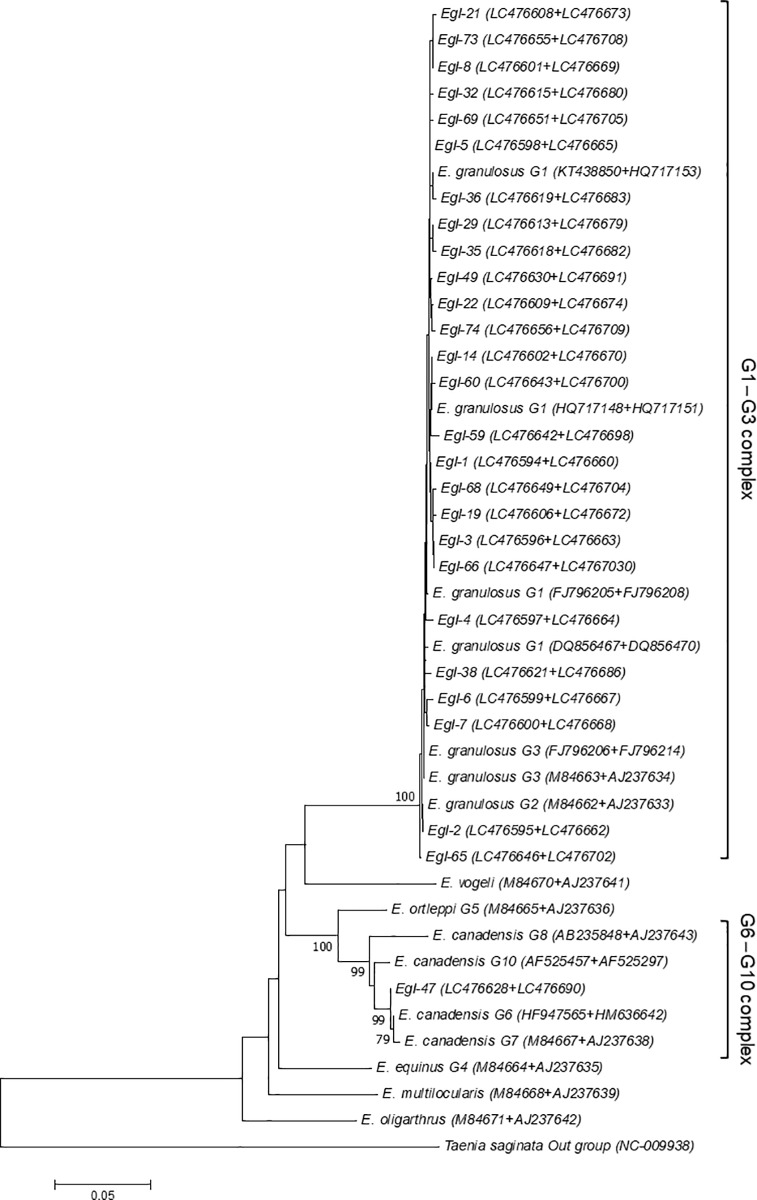
Phylogram of *Echinococcus granulosus* sensu lato was inferred based on the nucleotide sequences of concatenated cytochrome c oxidase I (*cox1*) and the NADH dehydrogenase subunit I (*nad1*). The evolutionary relationship of *Echinococcus granulosus* sensu lato constructed by the neighbor-joining method, based on the nucleotide sequences of concatenated *cox1* and *nad1* retrieved from this study (S1 Table) compared with reference sequences of *E*. *granulosus* sensu lato and other species of *Echinococcus* from GenBank, with *Taenia saginata* as outgroup. Bootstrap values obtained from 1000 replicates are indicated on branches in percentage, and only bootstraps values >70% are displayed. Evolutionary analyses were conducted in MEGA7.

The *cox1* and *nad1* fragments were successfully sequenced in 45 isolates. The sequencing data of each isolate were combined to produce the concatenated sequences. The alignment of 45 concatenated sequences revealed 28 haplotypes. Phylogenetic analysis showed 27 haplotypes clustered with published sequences representing genotypes G1-G3 and one with G6-G10, with strong bootstrap values (Fig 4).

## Discussion

Molecular analysis of sera and cyst tissue of patients with CE confirmed by surgery and histology detected *E*. *granulosus* DNA in 15.0% (95% CI: 8.8–24.4) and 10.0% (95% CI: 5.2–18.5) of serum samples based on the *cox1* and *nad1* gene, respectively. This finding may be a result of a low level of DNA filtration through the cyst wall. The DNA of *E*. *granulosus* may be more detectable in blood early in infection when the oncosphere is migrating through the circulatory system or when the cyst wall is not completely developed. However, patients undergoing CE surgery are usually in late stages with a large cyst having a thick impermeable wall that inhibits DNA release. Chaya and Parija [10] detected parasite DNA in serum in only 5 of 10 surgically confirmed CE cases in which the cyst was ruptured.

Both target mitochondrial genes in our study were amplified in large DNA fragments (400 and 450 bp), which might reduce the chance of detecting *E*. *granulosus* cell-free DNA (cfDNA) in serum. Due to the highly fragmented character of cfDNA [15, 16], it is predicted that the sensitivity of PCR might be improved by screening DNA fragments of 90–200 bp that are more likely to transfer through the cyst wall. Several studies have assessed cfDNA in serum, urine, or saliva as a diagnostic biomarker of infection with parasites [16] such as *Plasmodium spp*. [17, 18], *Entamoeba histocytica* [19], *Toxoplasma gondii* [20, 21], *Schistosoma* spp. [22–24], and *Strongyloides stercoralis* [25].

The quantity and quality of DNA are crucial to obtaining an accurate result in PCR. Among FFPE cyst samples of 80 CE patients, 91.3% (95% CI: 83.0–95.7) and 83.8% (95% CI: 74.2–90.3) were amplified, and 80.8% (95% CI: 70.3–88.2) and 74.6% (95% CI: 63.1–83.5) successfully sequenced for *cox1* and *nad1*, respectively. The obtained results from cyst tissue samples were in agreement with previous reports of 91.0% [26] by the *cox1* gene and 85.0% by the *nad*1 gene [27]. It is possible that formalin had increased DNA degradation in the non-amplified samples. Schneider et al. [27] stated that the sensitivity of single-round PCR can range from 35–85% in DNA extracted from FFPE tissues, depending on duration of storage in the paraffin block.

The sequencing and phylogenetic analyses revealed four genotypes of *E*. *granulosus* (G1, G2, G3, and G6) responsible for human surgically-treated CE in Iran, with genotype G1 predominating, followed by G3, G2, and G6. Genotype G1 is the most prevalent genotype worldwide [14, 26, 28–34], possibly due to the wide range of intermediate hosts, which facilitates higher circulation in the environment [35–37]. This phenomenon may be responsible for the high genetic diversity reported within genotype G1 [35], which was confirmed by our finding of 25 haplotypes.

In our samples, the most prevalent genotype after G1 was G3, in agreement with previous studies of human CE in Iran [31, 32] and various locations throughout the world [38]. The majority of reports of G3 are from Iran, India, and Italy [35, 38]. Kinkar et al. [38] suggested that distribution of genotype G3 [38] spread from Iran to India and Italy through domestic animal trade and that genotype G1 [35] similarly dispersed from Turkey to other parts of the world.

The least prevalent *E*. *granulosus* sensu stricto genotype, G2, is found worldwide [39, 40] with a few cases reported in livestock [41–43] and humans [26] in Iran. We found the G2 genotype by *cox1* sequencing analysis in three inhabitants of Tehran. Previous analysis of this locus has resulted in human G2 reported only in a single patient from Kerman [26]. The partial *nad1* gene sequence analysis was not able to distinguish between G2 and G3 in the fragment sequenced in the present study. This agrees with recent studies by Kinkar et al. [38, 44] who suggest that G2 is a microvariant of the G3 genotype and has not sufficiently mutated to qualify as a distinct mitochondrial genotype. This is supported by our phylogenetic tree based on concatenated sequences of *cox1* and *nad1* (Fig 4), in which the phylogram clusters do not support the separation of the G2 and G3 genotype sequences.

Genotype G6 was detected in one case of liver and one of lung CE. The results of this study agreed with the suggestion that, although genotype G6 is the second most common causative agent of human CE after the *E*. *granulosus* sensu stricto (G1-G3 complex) worldwide, its low occurrence in *E*. *granulosus* endemic areas exerts a minor influence on human health [36, 45]. However, it is the main cause of human CE in parts of the world in which animal infection by *E*. *granulosus* sensu stricto is rare [36]. Studies have shown that in the camel-rearing areas Kerman [26] and Birjand [46] of south-eastern and eastern Iran, genotype G6 is more prevalent than G1.

A limitation of this study was the identification of *E*. *granulosus* genotypes based on the partial *cox1* and *nad1* mitochondrial genes using sequences of insufficient length to separate the G1-G3 complex [35, 38, 44]. The short mitochondrial sequences were the optimal choice for amplifying low-quantity DNA in serum and relatively low-quality DNA in FFPE tissues exposed to formalin and are widely used for genotyping and phylogenetic studies of *E*. *granulosus*, providing a basis for comparing our findings.

## Conclusion

The finding of DNA specific to *E*. *granulosus* in 23.8% of serum samples from CE patients confirmed the presence of cfDNA released from the hydatid cyst. Although, due to the low quantity of detectable DNA in the serum, the test may be inadequate for the diagnosis of CE, it might be a starting point for further research into tracing smaller fragments of *E*. *granulosus* DNA to accelerate the diagnosis of the CE, particularly for screening high-risk individuals in endemic areas. The predominance of genotype G1 could confirm that the main transmission route of human infection is through the sheep-dog cycle.

## Supporting information

S1 Table Residence of patients undergoing cystic echinococcosis surgery, genotypes and GenBank accession numbers of *Echinococcus granulosus* identified in formalin-fixed paraffin embedded cyst tissue and serum by the cytochrome c oxidase I (*cox1*) and NADH dehydrogenase subunit I (*nad1*) mitochondrial genes. (DOCX)
