# Supplementary material for: Detection and genetic characterization of Echinococcus granulosus mitochondrial DNA in serum and formalin-fixed paraffin embedded cyst tissue samples of cystic echinococcosis patients
Source: PLoS One. 2019 Oct 29;14(10):e0224501. doi: 10.1371/journal.pone.0224501 (PMC6818807; doi:10.1371/journal.pone.0224501)
Supplement: S1 Table — (DOCX) [file pone.0224501.s001.docx]

**S1 Table**. Residence of patients undergoing cystic echinococcosis surgery, genotypes and GenBank accession numbers of *Echinococcus granulosus* identified in formalin-fixed paraffin embedded cyst tissue and serum by the cytochrome c oxidase I (*cox1*) and NADH dehydrogenase subunit I (*nad1*) mitochondrial genes.

| Isolate | Genotype | | City | Province | Accession Number | |
| --- | --- | --- | --- | --- | --- | --- |
|  | cox1 | nad1 |  |  | cox1 | nad1 |
| EgI-1 | G1 | G2/3 | Sanandaj | Kurdistan | LC476594 | LC476660 |
| EgI-1s* | G1 | G2/3 | Sanandaj | Kurdistan | ns | LC476661 |
| EgI-2 | G2 | G2/3 | Karaj | Alborz | LC476595 | LC476662 |
| EgI-3 | G1 | G2/3 | Pakdasht | Tehran | LC476596 | LC476663 |
| EgI-4 | G1 | G2/3 | Tehran | Tehran | LC476597 | LC476664 |
| EgI-5 | G1 | G1 | Tehran | Tehran | LC476598 | LC476665 |
| EgI-5s | G1 | G1 | Tehran | Tehran | ns | LC476666 |
| EgI-6 | G3 | G1 | Qarchak | Tehran | LC476599 | LC476667 |
| EgI-7 | G3 | G2/3 | Tehran | Tehran | LC476600 | LC476668 |
| EgI-8 | G1 | G1 | Mianeh | East Azerbaijan | LC476601 | LC476669 |
| EgI-14 | G1 | G1 | Takab | West Azerbaijan | LC476602 | LC476670 |
| EgI-15 | G1 | G2/3 | Karaj | Alborz | LC476603 | LC476671 |
| EgI-17 | G2 | ns | Tehran | Tehran | LC476604 | ns |
| EgI-18 | G1 | ns | Tehran | Tehran | LC476605 | ns |
| EgI-19 | G1 | G2/3 | Tehran | Tehran | LC476606 | LC476672 |
| EgI-20 | G1 | ns | Tehran | Tehran | LC476607 | ns |
| EgI-21 | G1 | G1 | Tehran | Tehran | LC476608 | LC476673 |
| EgI-22 | G1 | G1 | Mehrshahr | Alborz | LC476609 | LC476674 |
| EgI-23 | G1 | G1 | Karaj | Alborz | LC476610 | LC476675 |
| EgI-24 | G1 | G2/3 | Badreh | Ilam | LC476611 | LC476676 |
| EgI-25 | G1 | ns | Khodabandeh | Zanjan | LC476612 | ns |
| EgI-26 | ns | G1 | Tabriz | East Azerbaijan | ns | LC476677 |
| EgI-28 | ns | G1 | Tehran | Tehran | ns | LC476678 |
| EgI-29 | G1 | G1 | Varamin | Tehran | LC476613 | LC476679 |
| EgI-31 | G1 | ns | Nazarabad | Alborz | LC476614 | ns |
| EgI-32 | G1 | G1 | Tehran | Tehran | LC476615 | LC476680 |
| EgI-33 | G1 | G1 | Tehran | Tehran | LC476616 | LC476681 |
| EgI-34 | G3 | ns | Qazvin | Qazvin | LC476617 | ns |
| EgI-35 | G1 | G1 | Shahriar | Tehran | LC476618 | LC476682 |
| EgI-36 | G1 | G1 | Tehran | Tehran | LC476619 | LC476683 |
| EgI-37 | G1 | G1 | Saveh | Markazi | LC476620 | LC476684 |
| EgI-37s | ns | G1 | Saveh | Markazi | ns | LC476685 |
| EgI-38 | G3 | G1 | Tehran | Tehran | LC476621 | LC476686 |
| EgI-39 | G1 | ns | Saveh | Markazi | LC476622 | ns |
| EgI-41 | G1 | ns | Qods | Tehran | LC476623 | ns |
| EgI-42 | G1 | G1 | Varamin | Tehran | LC476624 | LC476687 |
| EgI-44 | G1 | ns | Shahriar | Tehran | LC476625 | ns |
| EgI-45 | G1 | G1 | Saveh | Markazi | LC476626 | LC476688 |
| EgI-46 | G1 | G1 | Qods | Tehran | LC476627 | LC476689 |
| EgI-47 | G6 | G6 | Qaemshahr | Mazandaran | LC476628 | LC476690 |
| EgI-48 | G1 | ns | Andisheh | Tehran | LC476629 | ns |
| EgI-49 | G1 | G1 | Tehran | Tehran | LC476630 | LC476691 |
| EgI-50 | G1 | G1 | Tehran | Tehran | LC476631 | LC476692 |
| EgI-51 | G1 | ns | Tehran | Tehran | LC476632 | ns |
| EgI-51s | G1 | ns | Tehran | Tehran | LC476633 | ns |
| EgI-52 | ns | G1 | Tehran | Tehran | ns | LC476693 |
| EgI-53 | G6 | G6 | Tehran | Tehran | LC476634 | LC476694 |
| EgI-54 | G1 | G1 | Tehran | Tehran | LC476635 | LC476695 |
| EgI-54s | G1 | ns | Tehran | Tehran | LC476636 | ns |
| EgI-55 | G1 | G1 | Tehran | Tehran | LC476637 | LC476696 |
| EgI-56 | G1 | ns | Malard | Tehran | LC476638 | ns |
| EgI-56s | G1 | ns | Malard | Tehran | LC476639 | ns |
| EgI-57 | G1 | G2/3 | Qods | Tehran | LC476640 | LC476697 |
| EgI-57s | G1 | ns | Qods | Tehran | LC476641 | ns |
| EgI-59 | G1 | G1 | Tehran | Tehran | LC476642 | LC476698 |
| EgI-59s | ns | G1 | Tehran | Tehran | ns | LC476699 |
| EgI-60 | G1 | G1 | Tehran | Tehran | LC476643 | LC476700 |
| EgI-61 | G1 | G1 | Nasimshahr | Tehran | LC476644 | LC476701 |
| EgI-62 | G1 | ns | Tehran | Tehran | LC476645 | ns |
| EgI-65 | G2 | G2/3 | Tehran | Tehran | LC476646 | LC476702 |
| EgI-66 | G1 | G2/3 | Tehran | Tehran | LC476647 | LC476703 |
| EgI-67 | G1 | ns | Eslamshahr | Tehran | LC476648 | ns |
| EgI-68 | G1 | G2/3 | Qods | Tehran | LC476649 | LC476704 |
| EgI-68s | G1 | ns | Qods | Tehran | LC476650 | ns |
| EgI-69 | G1 | G1 | Tehran | Tehran | LC476651 | LC476705 |
| EgI-70 | G1 | G2/3 | Qazvin | Qazvin | LC476652 | LC476706 |
| EgI-70s | G1 | ns | Qazvin | Qazvin | LC476653 | ns |
| EgI-72 | G1 | G1 | Shahriar | Tehran | LC476654 | LC476707 |
| EgI-73 | G1 | G1 | Tehran | Tehran | LC476655 | LC476708 |
| EgI-74 | G1 | G1 | Andisheh | Tehran | LC476656 | LC476709 |
| EgI-74s | G1 | ns | Andisheh | Tehran | LC476657 | ns |
| EgI-75 | G1 | G1 | Tehran | Tehran | LC476658 | LC476710 |
| EgI-76 | ns | G1 | Tehran | Tehran | ns | LC476711 |
| EgI-77 | ns | G1 | Tehran | Tehran | ns | LC476712 |
| EgI-78 | G1 | G1 | Tehran | Tehran | LC476659 | LC476713 |
| EgI-79 | ns | G1 | Tehran | Tehran | ns | LC476714 |

ns: not successfully sequenced.

*s: relative to the serum sample.
